# CAT: Class-aware adaptive-thresholding for robust semi-supervised domain generalization

**DOI:** 10.1371/journal.pone.0329799

**Published:** 2025-09-04

**Authors:** Sumaiya Zoha, Jeong-Gun Lee, Young-Woong Ko

**Affiliations:** 1 Department of Computer Science and Engineering, Ahsanullah University of Science and Technology, Dhaka, Bangladesh; 2 Department of Computer Engineering, Hallym University, Chuncheon, South Korea; Kafkas University: Kafkas Universitesi, TÜRKIYE

## Abstract

Domain Generalization (DG) seeks to transfer knowledge from multiple source domains to unseen target domains, even in the presence of domain shifts. Achieving effective generalization typically requires a large and diverse set of labeled source data to learn robust representations that can generalize to new, unseen domains. However, obtaining such high-quality labeled data is often costly and labor-intensive, limiting the practical applicability of DG. To address this, we investigate a more practical and challenging problem: semi-supervised domain generalization (SSDG) under a label-efficient paradigm. In this paper, we propose a novel method, **CAT**, which leverages semi-supervised learning with limited labeled data to achieve competitive generalization performance under domain shifts. Our method addresses key limitations of previous approaches, such as reliance on fixed thresholds and sensitivity to noisy pseudo-labels. **CAT** combines adaptive thresholding with noisy label refinement techniques, creating a straightforward yet highly effective solution for SSDG tasks. Specifically, our approach uses flexible thresholding to generate high-quality pseudo-labels with higher class diversity while refining noisy pseudo-labels to improve their reliability. Extensive experiments on multiple benchmark datasets demonstrate the superior performance of our method, with improvements of **3.45%** on *PACS*, **9.47%** on *OfficeHome*, and **10.90%** on *miniDomainNet* datasets, highlighting its effectiveness in achieving robust generalization under domain shifts.

## 1 Introduction

**Fig 1:**
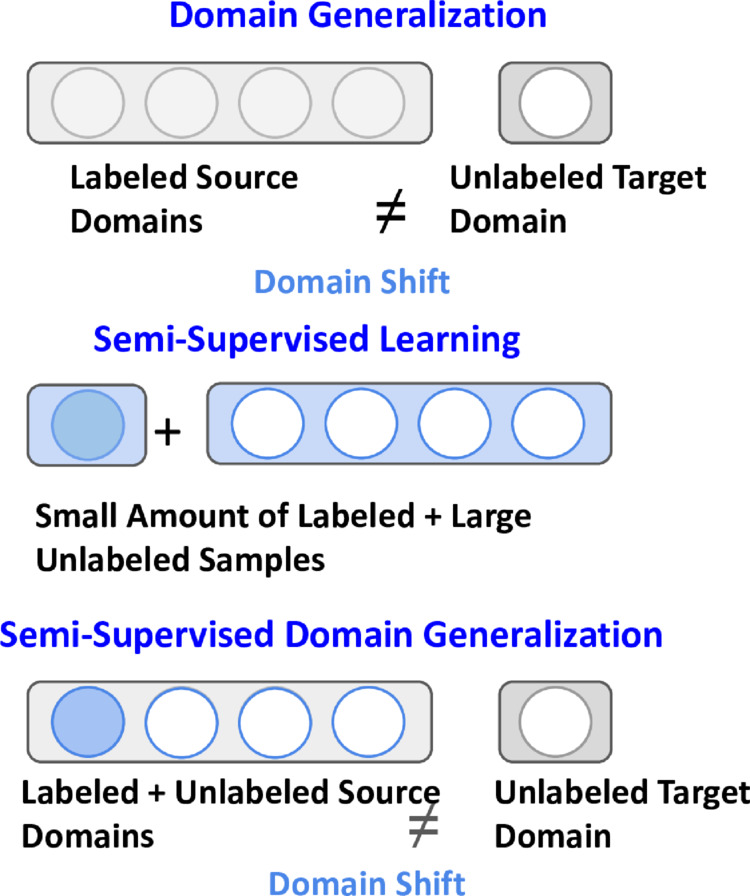
In typical domain generalization setting, multiple labeled domains are used for training, where as in semi-supervised learning, a few annotated data are used with a large amount of annotated data. But in semi-supervised domain generalization (SSDG), a few annotated data used with a large amount of labeled data from multiple domains.

Most machine learning models assume that the train and test data are drawn from the same distribution, but in practice, both distributions can frequently change due to the involving test distribution. For this limitation, these models cannot be generalized in real-world applications, such as autonomous driving systems or medical applications [1–3]. On the other side, deep neural networks have shown remarkable success in various classification tasks under fully annotated training conditions. Most deep learning (DL) models require a large amount of labeled data to achieve comparable results. However, in real-world applications, collecting labeled data is challenging due to its substantial cost and the need for human annotation [4–7]. Recently, semi-supervised learning (SSL) [4,6,8] techniques have gained significant attention for their ability to effectively utilize unlabeled data alongside a small amount of labeled data. The main challenge in SSL lies in learning effective representations of unlabeled data in relation to labeled examples to enhance generalization performance. To address this, techniques such as pseudo-labeling [9–11] and consistency regularization [12–14] have proven to be effective. However, these methods are designed primarily for single-source classification tasks, making it difficult for them to capture multiple cross-domain relationships, a critical requirement for domain generalization (DG). Please refer to Fig 1 for illustration of DG, SSL and semi-supervised domain generalization (SSDG).

Domain shift [15–17] presents a significant challenge in deploying deep learning models, especially in critical applications such as medical imaging and self-driving systems, where domain shifts can lead to severe risks. To address this, domain generalization (DG) methods have been developed [18–21]. Most DG methods rely on supervised learning, where a model is trained on multiple labeled source domains. However, in real-world scenarios, obtaining sufficient labeled data for these domains is often impractical and burdensome.

On the other hand, unlabeled samples from source domains are more feasible and abundant. The challenge lies in their variability and the presence of unknown classes. Most SSL methods leverage these abundant unlabeled samples with the guidance of labeled samples to generate pseudo-labels. Producing accurate pseudo-labels is essential for effectively utilizing unlabeled data in model training. Nevertheless, existing DG methods heavily depend on fully annotated source samples to perform well, limiting their applicability in real-world scenarios. In this paper, we explore the potential of the SSL paradigm in DG settings, referred to as semi-supervised domain generalization (SSDG). Fig 1 illustrates the differences between SSL, DG and SSDG methods.

As described above, pseudo-labeling is effective for utilizing unlabeled samples, but many methods rely on fixed thresholding. For example, FixMatch [13] uses a fixed threshold for all classes, which often discards too many unlabeled samples with correct pseudo-labels. In SSDG settings, StyleMatch [22] extends the same fixed-threshold strategy as FixMatch [13], but its performance is similarly limited by the loss of valuable unlabeled samples. Adaptive and dynamic class-dependent thresholding offers a reliable solution to this issue [23–25]. However, these methods are designed for single-domain SSL settings, making multi-domain training—a strict requirement for DG—challenging and often infeasible for achieving successful SSDG.

Our work is closely related to recent advances in SSL and DG. FreeMatch [23] addresses this limitation by introducing a self-adaptive thresholding mechanism based on the model’s learning status, while our approach is inspired from FreeMatch, we extend its thresholding mechanism to the multi-domain setting by introducing *class-domain adaptive thresholds*. This allows our method to better capture both class-specific and domain-specific variations in the data, which is essential for robust pseudo-labeling under domain shift. Moreover, AdaMatch [26] presents a unified approach to SSL, and unsupervised domain adaptation (UDA), by aligning distributions of weak and strong augmentations and calibrating confidence thresholds using batch statistics. Unlike AdaMatch, which assumes access to unlabeled target data for adaptation, our method tackles the more challenging domain generalization problem where target domain data is completely unseen during training. Finally, PCL [27] finds that naive application of supervised contrastive learning can degrade generalization due to misaligned sample pairs across domains. To mitigate this, PCL proposes proxy-based contrastive learning to align representations via learned prototypes. We instead retain sample-to-sample contrastive learning but improve it by first identifying noisy pseudo-labeled samples. This enables robust representation learning without explicit proxy usage. Our method also complements this with unsupervised contrastive learning on uncertain samples, allowing us to refine noisy pseudo-labels while preserving domain diversity and structure.

To address these limitations, we propose **CAT**, an adaptive thresholding method specifically designed for SSDG settings. CAT overcomes the drawbacks of fixed-threshold approaches by employing adaptive class-dependent thresholds tailored for SSDG tasks. We utilize both global and local thresholds, iteratively increasing the thresholds based on the training time steps. This strategy allows the model to capture more correct pseudo-labels compared to strictly fixed thresholds. Local thresholding is employed to ensure variability across class labels and to improve the confidence dynamics for producing pseudo-labels. In parallel, a noisy label refinement module is integrated to further refine pseudo-labels, ensuring higher quality. Additionally, we leverage supervised contrastive learning with the refined pseudo-labels to achieve domain-invariant representations. Experimental results on several benchmarks demonstrate the superiority of our method. Our contributions are threefold:

Our work is closely related to recent advances in SSL and DG. FreeMatch [23] addresses this limitation by introducing a self-adaptive thresholding mechanism based on the model’s learning status, while our approach is inspired from FreeMatch, we extend its thresholding mechanism to the multi-domain setting by introducing *class-domain adaptive thresholds*. This allows our method to better capture both class-specific and domain-specific variations in the data, which is essential for robust pseudo-labeling under domain shift. Moreover, AdaMatch [26] presents a unified approach to SSL, and unsupervised domain adaptation (UDA), by aligning distributions of weak and strong augmentations and calibrating confidence thresholds using batch statistics. Unlike AdaMatch, which assumes access to unlabeled target data for adaptation, our method tackles the more challenging domain generalization problem where target domain data is completely unseen during training. Finally, PCL [27] finds that naive application of supervised contrastive learning can degrade generalization due to misaligned sample pairs across domains. To mitigate this, PCL proposes proxy-based contrastive learning to align representations via learned prototypes. We instead retain sample-to-sample contrastive learning but improve it by first identifying noisy pseudo-labeled samples. This enables robust representation learning without explicit proxy usage. Our method also complements this with unsupervised contrastive learning on uncertain samples, allowing us to refine noisy pseudo-labels while preserving domain diversity and structure.

- Motivated by the challenges of generating high-quality pseudo-labels for SSDG, we propose a method that produces robust pseudo-labels, effectively mitigating the impact of noise.
- We introduce **CAT**, a simple yet effective approach that integrates adaptive thresholding with a noisy label refinement module to achieve superior performance in SSDG settings.
- Extensive experiments on multiple benchmarks validate the effectiveness of our method. **CAT** not only outperforms state-of-the-art SSDG methods but also surpasses standalone DG and SSL approaches.

The organization of this paper is as follows: Sect 2 presents related work that comprehensively reviewed relevant works, Sect 3 details our proposed method, Sect 4 presents the experimental results, demonstrating the effectiveness of our approach comparing with other state-of-the-art methods, and Sect 5 concludes the paper by summarizing the findings and providing future directions.

## 2 Related works

**Domain generalization.** Domain generalization (DG) aims train with multiple source domains and transfer to unseen target domains. Most DG settings consider source and target domains to be from different distributions. The main goal is to perform well under this distribution shift, also called domain shift. DG can be categorized into multiple methods such as domain alignment, meta-learning, adversarial learning, data augmentation, ensemble learning, self-supervised learning, and feature regularization [18]. Domain alignment methods are based on minimizing moments [28], KL-divergence [29], and maximum mean discrepancy [30] to learn domain-invariant representations. In meta-learning-based DG, training data is divided into meta-train and meta-test sets to improve generalization on the meta-test set. Most existing methods are based on episode construction, where source domains are divided into meta-train and meta-test domains to stimulate domain shift [31,32]. Other prominent approaches, such as adversarial learning where the learned features are enforced to be agnostic to domain information [30,33]. In augmentation, most of the works are related to feature augmentation [20,34,35] or model-based augmentation [36]. Ensemble learning techniques learn multiple models with different initializations and utilize their ensemble for prediction, examples are domain-specific neural networks [37,38] and batch normalization [39,40]. Self-supervised learning explores pretext tasks that allow a model to learn invariant features [41,42]. Lastly, regularization methods are based on feature regularization [43] and model regularization [44].

**Semi-supervised learning.** Semi-supervised learning (SSL) refers to learning from limited data and utilizing abundant unlabeled data. SSL aims to predict data accurately assuming that labeled and unlabeled data are from an identical distribution [8,9,45]. Most SSL techniques are based on pseudo-labels [9,10], mean-teacher [46–48], and consistency regularization [12–14]. Except for consistency regularization, entropy-based regularization is also widely used in SSL, where entropy minimization encourages the model to make confident predictions based on all samples [49]. On the other hand, thresholding-based methods FixMatch [13], FreeMatch [23], and UDA [50] select samples based on pre-defined thresholds during training, so multiple works proposed adaptive and dynamic thresholding to alleviate this limitation. DASH [25], AdaMatch [26] uses a pre-defined threshold to adjust based on the loss from labeled data and multiply average confidence to noisy pseudo labels. Self-training [51–53] methods are also effective in SSL settings, it is also known as decision-directed learning where the main goal is to determine the decision boundary on low-density regions [54].

**Semi-supervised domain generalization (SSDG).** Semi-supervised domain generalization (SSDG) involves SSL and DG which is a more difficult setting due to utilizing a large amount of unlabeled data to achieve competitive DG results. One most recent works is StyleMatch [22], which utilizes a stochastic classifier to extend FixMatch [13] with multi-view consistency to achieve SSDG. Another line of work is based on utilizing known and unknown classes with class-adaptive method [55]. MultiMatch [56] extends FixMatch [13] but in a multi-task setting by producing high-quality pseudo-labels for SSDG. Although these methods achieved comparable results in SSDG tasks, but not sufficient for real-world practicability. In Table 1, we provided a comparison between our work and other related works such as StyleMatch [22] and MultiMatch [56].

**Table 1:** Comparison with related semi-supervised domain generalization methods.

Method	Year	Uses Pseudo-Labels	Adaptive Thresholding	Refines Noisy Labels	Key Differentiator
StyleMatch [22]	2023	✓	**✘**	**✘**	Relies on FixMatch with a single, fixed confidence threshold for all classes and domains.
MultiMatch [56]	2024	✓	**✘**	**✘**	Focuses on multi-view consistency but typically without dynamic thresholds or explicit noise refinement.
**CAT (Ours)**	2025	✓	✓	✓	Employs dynamic, class-aware thresholds, an explicit module for refining noisy pseudo-labels, and uses these refined labels to guide supervised contrastive learning.

## 3 Method

**Fig 2:**
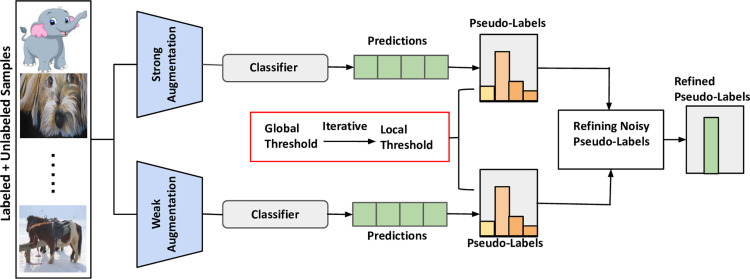
The overall pipeline of CAT. Given a few labeled and unlabeled samples from multiple source domains, CAT first performs weak and strong augmentations to get initial pseudo-labels. Then, CAT employs global and local thresholds that can dynamically adapt thresholding based on class dependency. Finally, noisy pseudo-label refining module is employed to get noise-free pseudo-labels.

Our method **CAT**, is a semi-supervised domain generalization (SSDG) approach that considers dynamically adjusting class-dependent thresholds using global and local thresholding, iteratively increasing them over training steps. This dynamic thresholding approach helps to capture accurate pseudo-labels with fewer errors; on the other hand, local thresholding enables variability across class-labels, improving confidence and class-wise pseudo-label quality. To further refine pseudo-labels, we integrate a noisy label refinement module, which filters out low-quality labels and ensures higher reliability. Additionally, a supervised contrastive learning approach with refined pseudo-labels is employed to learn domain-invariant representations. An overview of our method is given in Fig 2.

### 3.1 Notation and preliminaries

**Semi-supervised learning.** In SSL settings, we are given a set of *N* labeled samples from an unknown distribution, which includes sample and label pairs ${𝒟}_{L}=\{(x_{i},y_{i}){\}}_{i=1}^{N}$, and *M* unlabeled samples without defined labels ${𝒟}_{U}=\{(x_{i}){\}}_{i=1}^{M}$. There are *k* classes, where *N*_*k*_ and *M*_*k*_ are the numbers of labeled and unlabeled samples in the *k*-th class, respectively. Without loss of generality, $M_{k}\gg N_{k}$. The training loss calculated in an SSL algorithm usually contains a supervised loss ${ℒ}_{s}$ and an unsupervised loss ${ℒ}_{u}$. Typically, ${ℒ}_{s}$ is calculated based on ${𝒟}_{L}$ samples with a cross-entropy loss. The loss function is defined as:

$${ℒ}_{s}=\frac{1}{N}\sum_{i=1}^{N}H(y_{i},f(𝐲|{𝐱}_{i};\theta ))$$ (1)

Expanding the entropy term:


$${ℒ}_{s}=\frac{1}{N}\sum_{i=1}^{N}\sum_{k=1}^{K}-y_{i,k}\log f(𝐲=k|𝐱;\theta )$$

Here, $f(𝐲|𝐱;\theta )\in [0,1{]}^{K}$ is the probabilities produced by the model function *f*, which is parameterized by *θ* for the input **x**, and H(·,·) is the cross-entropy loss. The unsupervised loss ${ℒ}_{u}$ is calculated based on different settings of SSL algorithms. One key example is from FixMatch [13], where the unsupervised loss is guided by generating pseudo-labels, and eventually using the same supervised loss objective via cross-entropy loss.

**Domain generalization.** In typical DG settings, we have *k* source domains, each containing *N* samples. The inputs **x** and their corresponding **y** labels are drawn from a joint distribution. The *k* source domains are similar but distinct, denoted as ${𝒟}_{S}=\{(x_{i},y_{i}){\}}_{i=1}^{N}$. The main goal of DG is to learn a model function *f* that can leverage these *k* sources to learn a representation that performs well on unlabeled and unseen target samples ${𝒟}_{T}=\{x_{i}\}$, by reducing the domain shift between the source and target domains.

$$\min_{h}\;{𝔼}_{(𝐱,y)\in {𝒟}_{T}}[ℒ(h(𝐱),y)]$$ (2)

Here, 𝔼 represents the expectation and ℒ(·,·) is the loss function.

**Semi-supervised domain generalization.** Similar to the conventional DG setting, we have multiple diverse domains ${𝒟}_{k}$ from *k* source domains, where each source domain ${𝒟}_{L}=(x_{i},y_{i})$ consists of pairs of images and corresponding labels [22,57]. However, in the SSDG setting, each source domain contains only a small number of labeled samples $n_{L}\in [5,10]$, while the remaining labels are unlabeled, denoted as *n*_*U*_, with $n_{U}\gg n_{L}$ in each source domain. This setting combines aspects of both SSL and DG. The ultimate goal is to learn a domain-generalizable model using both labeled and unlabeled source data ${𝒟}_{S}=\{n_{U}\cup n_{L}\}$, such that the model performs well on unseen target data. A summary of notations is given in Table 2.

**Table 2:** Summary of notations.

Notation	Description
${𝒟}_{L}$	Set of labeled data samples $\left(x_{i},y_{i}\right)$
${𝒟}_{U}$	Set of unlabeled data samples *x*_*i*_
*N*	Number of labeled samples
*M*	Number of unlabeled samples
*K* or *C*	Number of classes
f(·;θ)	Model function parameterized by *θ*
${ℒ}_{s}$	Supervised loss (cross-entropy on labeled data)
${ℒ}_{u}$	Unsupervised loss (based on pseudo-labels)
${ℒ}_{\text{scl}}$	Supervised contrastive loss
${ℒ}_{T}$	Total loss: ${ℒ}_{s}+\lambda_{u}{ℒ}_{u}+{ℒ}_{\text{scl}}$
${𝐪}_{i}$	True label distribution (one-hot for labeled data)
$p_{k}(\cdot )$	Predicted probability distribution over class *k*
*τ*	Threshold for confidence-based pseudo-labeling
$\tau_{g}$	Global confidence threshold
$\tau_{g}(c)$	Class-specific global threshold
*λ*	Momentum decay parameter in EMA
μB	Batch size for unlabeled data (with multiplier *μ*)
${𝐳}_{i}$	Representation of sample *i* in contrastive learning
*G*(*i*)	Positive sample set for anchor *i*
*A*(*i*)	All samples used for contrastive comparison with *i*
*τ*	Temperature parameter in contrastive loss
d(𝐚,𝐛)	Cosine similarity between vectors a and b
${ℰ}_{t}$	EMA of confidence scores at step *t*
*P*	Set of low-noise samples selected for contrastive learning
*α*	Fractile threshold for pseudo-label confidence agreement
*n*_*L*_	Number of labeled samples per source domain
*n*_*U*_	Number of unlabeled samples per source domain
${𝒟}_{S}$	Source domain dataset
${𝒟}_{T}$	Target domain dataset (unseen during training)

### 3.2 Class-domain aware thresholding

Due to its simplicity and effectiveness, StyleMatch [22] leverages FixMatch [13] to generate pseudo-labels using a classifier with a fixed threshold. In this work, we revisit FixMatch to understand better the process of selecting unlabeled candidate samples for pseudo-label generation, particularly the fixed confidence threshold. We argue that relying on a fixed threshold may exclude a significant number of unlabeled samples that could receive accurate pseudo-labels, thereby limiting the practical applicability of FixMatch in data-efficient scenarios. Another challenge is that these thresholds are not class-independent, which makes FixMatch less suited for capturing class-variant information, especially in multi-domain settings. In FixMatch [13], supervised loss ${ℒ}_{s}$ and unsupervised loss ${ℒ}_{u}$ are employed for labeled and unlabeled data, respectively, where ${ℒ}_{s}$ corresponds to the standard cross-entropy loss:

$${ℒ}_{s}=\frac{1}{N}\sum_{i=1}^{N}ℋ({𝐪}_{i},p_{k}(g({𝐱}_{i}^{l});\phi )),$$ (3)

Here, *N* denotes the number of samples, and ℋ(.) represents the loss function, where the true distribution ${𝐪}_{i}$ and the predicted distribution *p*_*k*_ are provided. Motivated by the limitations of FixMatch [13] in generating pseudo-labels, we focus on adaptive thresholding, which is less restrictive and more flexible in selecting class-wise samples. Recently, adaptive and dynamic thresholding methods have demonstrated effectiveness in SSL settings [23–25], primarily due to their ability to handle class-dependent samples flexibly. However, in the DG it is crucial not only to adaptively select class-dependent samples but also to preserve domain-specific information. This dual requirement is essential for leveraging unlabeled data effectively while maintaining domain and class consistency. Unlike prior methods such as [23,24], which adaptively set class-dependent thresholds without considering domain-specific information, we propose a method that incorporates both class and domain dependencies in pseudo-label selection. In FreeMatch [23], global and local thresholds are set to be both dataset- and class-specific. Inspired by this approach, we extend the concept to simultaneously define domain- and class-dependent thresholds. By incorporating these dual thresholds, our method dynamically selects pseudo-labels based on both class and domain information, thereby maximizing the utility of unlabeled samples in the DG setting.

**Data augmentation.** We use UDA [50] strategy for data augmentation to get weak and strong augmentation. Inspired by FixMatch [13] and FreeMatch [23], we use RandAugment [58] for strong augmentation. Data augmentation is used for retaining pseudo-labels on the unlabeled data followed by an unsupervised loss [23]:

$${ℒ}_{u}=\frac{1}{\mu B}\sum_{b=1}^{\mu B}ℋ(\;⊮(\max({𝐪}_{b})>\tau ),p_{k}(g({𝐱}_{b}^{u});\phi ))$$ (4)

Here, (⊮) is the indicator function for confidence-based thresholding [13].

**Class-specific global and local thresholding.** Following [23], we utilize a global threshold to iteratively increase the threshold to engage with many samples with a low threshold, then it stably discards incorrect pseudo labels. Based on the *t*–th time step, the model’s average confidence on the unlabeled data to compute the global threshold $\tau_{g}$. $\tau_{g}$ is initialized as 1/*C* where *C* is the number of class in each source domain ${𝒟}_{S}$. Then $\tau_{g}$ is adjusted in each time step *t* [23] based on the exponential moving average (EMA):

$$\tau_{g}=\left\{ \begin{array}{cc} \frac{1}{C}, & \text{if }t=0,\\ \lambda \tau_{t-1}+\frac{\left(1-\lambda \right)}{\mu B}\sum_{b=1}^{\mu B}[\max(q_{b})], & \text{if }t>0. \end{array}\right.$$ (5)

Here, λ∈{0,1} is the momentum decay of EMA. Now, to adjust the global threshold in a class-specific manner. The expectation of the model’s prediction on each class *c* based on the source domain ${𝒟}_{S}$ to estimate class-specific learning.

$${ℰ}_{t}=\left\{ \begin{array}{cc} \frac{1}{C}, & \text{if }t=0,\\ \lambda {ℰ}_{t-1}+\frac{\left(1-\lambda \right)}{\mu B}\sum_{b=1}^{\mu B}[\max(q_{b})], & \text{if }t>0. \end{array}\right.$$ (6)

Here, ${ℰ}_{t}=[{ℰ}_{t}(1),.......{ℰ}_{t}(C)]$ is the list of all existing classes. Then we integrate Max Normalization to obtain a self-adaptive threshold based on each class $\tau_{g}(c)$.

$$\tau_{g}(c)=\text{MaxNorm}({ℰ}_{t}(c)).\tau_{g}$$ (7)

So, the final unsupervised loss can be formulated as [23]:

$${ℒ}_{u}=\frac{1}{\mu B}\sum_{b=1}^{\mu B}ℋ(\;⊮(\max({𝐪}_{b})>\tau_{g}(\text{argmax}(q_{b})),p_{k}(g({𝐱}_{b}^{u});\phi ))$$ (8)

### 3.3 Refining noisy pseudo labels

Contrastive learning (CL) aims to learn universal prior information that can be applied to downstream tasks. In this approach, we use CL to extract universal prior knowledge from positive and negative samples and leverage it to enhance generalization performance in downstream tasks [59]. In CL, a common strategy is to pull positive pairs (which are semantically similar) closer together and push negative pairs (which are semantically dissimilar) farther apart. Conventional CL methods are related to leverage unlabeled samples, with unsupervised fashion. But based on the pseudo labeled based on self-adaptive thresholding for the unlabeled samples, we construct positive and negative samples based on supervised CL [59]. Where we consider labeled information is available. But obtained pseudo-labels can be noisy that can lead to poor generalization performance. This enhances multi-domain learning and allows understanding of the class-specific samples to sample relationships from diverse domains from the source dataset. To enable multi-domain learning, we utilize supervised contrastive learning assuming some of the pseudo labels can be noisy that can affect the generalization performance, which can align these hard samples, inspired by [27]. We use unsupervised-CL for warm up training where low-dimentional representation and pseudo-labels are given. Our goal is to find the similarity of the given samples by using cosine distance.

$$d(𝐚,𝐛)=\frac{𝐚\cdot {𝐛}^{⊤}}{\left\|𝐚\|\|𝐛\right\|}$$ (9)

Where, a,b are the low-dimensional representations. For each sample given by its pseudo labels $\left(x_{i},\hat{y_{i}}\right)$, we aggregate its original label based on the top-*K* neighbors based on the similarity of their representations. In this way, we can improve the detection of mislabeled pseudo-labeled samples. To achieve more confident labels, we use the *α* fractile based on per class, which gives the agreements between the corrected labels based on the neighbors and similarity and original pseudo-labels across all classes [60,61]. After identifying the less noisy samples, we construct a set 𝒫 for representation learning. This set also help us to indentify whether given two instances belong from a same class or not.

**Supervised contrastive learning.** We use supervised CL loss that can handle the presence of labels, where supervised loss considers all samples from the same class as positive, and rest of the remaining samples as negative. This loss can enhance the representation learning from the given less noisy 𝒫 samples. The supervised CL objective can be written as:

$${ℒ}_{scl}=\sum_{i\in I}\frac{1}{\left|𝒫(i)\right|}\sum_{g\in G(i)}\log\frac{\exp({𝐳}_{i}\cdot {𝐳}_{p}/\tau )}{\sum_{a\in A(i)}\exp({𝐳}_{i}\cdot {𝐳}_{n}/\tau )}$$ (10)

Here, τ∈ℛ+ is a temperature parameter. Despite using supervised loss in the less noisy samples, we perform unsupervised CL on rest of the unselected samples, following [61].

**Final training objective.** Lastly, combining all losses, we can obtain the final loss ${ℒ}_{T}$ such as:

$${ℒ}_{T}={ℒ}_{s}+\lambda_{u}{ℒ}_{u}+{ℒ}_{scl}$$ (11)

Where, $\lambda_{u}$ represents the loss weight for ${ℒ}_{u}$.

**Algorithm 1. CAT.**

1: **Input:** Labeled dataset ${𝒟}_{L}$, unlabeled dataset ${𝒟}_{U}$, model *f* with parameters *θ*, batch size *B*, confidence threshold $\tau_{g}$

2: **Initialize:** Model parameters *θ*, EMA decay *λ*, class-specific confidence scores ${ℰ}_{0}=\frac{1}{C}$

3: **for** each training step t **do**

4:   Sample labeled batch $(x_{i},y_{i})\sim {𝒟}_{L}$ of size B

5:   Sample unlabeled batch $x_{j}\sim {𝒟}_{U}$ of size μB

{Compute supervised loss}

6:   ${ℒ}_{s}=\frac{1}{B}\sum_{i=1}^{B}H(y_{i},f(x_{i};\theta ))$

{Generate pseudo-labels for unlabeled data}

7:   **for** each *x*_*j*_ in unlabeled batch **do**

8:    $p_{j}=f(x_{j};\theta )$

9:    ${\hat{y}}_{j}=\arg\max(p_{j})$

10:    **if** $\max(p_{j})>\tau_{g}({\hat{y}}_{j})$ **then** retain ${\hat{y}}_{j}$

11:   **end for**

{Compute unsupervised loss}

12:   ${ℒ}_{u}=\frac{1}{\mu B}\sum_{j=1}^{\mu B}H(⊮(\max(p_{j})>\tau_{g}({\hat{y}}_{j})),p_{j})$

{Update global threshold using EMA}

13:   $\tau_{g}=\lambda \tau_{t-1}+(1-\lambda )\frac{1}{\mu B}\sum_{j=1}^{\mu B}\max(p_{j})$

{Class-specific thresholding}

14:   ${ℰ}_{t}=\lambda {ℰ}_{t-1}+(1-\lambda )\frac{1}{\mu B}\sum_{j=1}^{\mu B}\max(p_{j})$

15:   $\tau_{g}(c)=\text{MaxNorm}({ℰ}_{t}(c))\cdot \tau_{g}$

{Update model parameters}

16:   $\theta \leftarrow \theta -\eta \nabla_{\theta }({ℒ}_{s}+{ℒ}_{u})$

17: **end for**

18: **Return:** Trained model f with parameters *θ*

## 4 Experimental settings

### 4.1 Datasets

We use three publicly available datasets such as PCAS [21], OfficeHome [62], VLCS [63] and miniDomainNet [20] to evaluate our model with other baselines for semi-supervised domain generalization tasks. **PACS** contains 7 classes of images from distinct 4 domains (Photo - P, Art Painting - A, Cartoon - C and Sketch - S), **OfficeHome** contains images from 4 different domains (Artistic - A, Clip art - C, Product - P and Real-world - R). It is a relatively large dataset with 65 distinct classes related to daily life objects found in offices and homes. We also use **miniDomainNet**. It is a subset dataset of DomainNet with 4 different domains (Clipart - C, Painting - P, Real - R and Sketch - S), it covers almost 126 distinct classes. We report the average accuracy over the last five epochs as the final results. A summary of information about the datasets is given in Table 3.

**Table 3:** Summary of PACS, OfficeHome, VLCS, and miniDomainNet datasets, including the number of samples, domains, and domain names.

Dataset	# Samples	# Domains	Domain Names
**PACS**	9,991	4	Photo, Art Painting, Cartoon, Sketch
**OfficeHome**	15,500	4	Art, Clipart, Product, Real World
**miniDomainNet**	140,006	4	Clipart, Painting, Real, Sketch

### 4.2 Implementation details

We followed the protocol described in [21,22], these are common practice protocols in domain generalization setting. We utilize the leave-one-domain-out method, in which the model is trained with *n*–1 number of domains from the training dataset and evaluated on the remaining domain [21]. Pre-trained ResNet-18 and ResNet-50 variants [64] are used as the backbone of the model. Following [22], we randomly sample 16 images from the source domain for the mini-batch reconstruction with labeled and unlabeled data. With guidance from the labeled data, we generate the pseudo and proxy labels using the unlabeled data. The learning rate is set to 0.003, we examined multiple learning rates to find the best one. We use SGD optimizer with a standard momentum 0.9, and epochs are 40, 20, 20 for PACS, OfficeHome and miniDomainNet respectively with early stopping based on the validation accuracy (patience every 5 epochs). All models are trained using an RTX 3090 GPU. Our implementation is based on Dassl.pytorch [20] toolbox. For data preprocessing, we follow the standard practice used in [21,22], input images are resized to 224×224, and augmented with random cropping and horizontal flipping. We use the SGD optimizer with a standard momentum 0.9, and epochs are 40, 20, 20 for PACS, OfficeHome, and miniDomainNet, respectively, with early stopping based on the validation accuracy (patience every 5 epochs). We set $\lambda_{u}$ = 1 for all experimental cases. We use the official train-validate split of each dataset for validation, and labeled samples are randomly sampled from the training split. All samples of the target domain are used as test data.

## 5 Experimental results

### 5.1 Comparison with state-of-the-art methods

In this experiment, we compare our method with multiple state-of-the-art methods on standard DG datasets to verify the effectiveness of our method. We divide the comparison with four different paradigms (*i.e.* fully-labeled, domain generalization methods, semi-supervised methods, and semi-supervised domain generalization method). In the fully labeled setting, all source labels are available during training under the conventional DG settings. In the DG setting, we compare our method with vanilla training, CrossGrad [65], DDAIG [66], RSC [67] and EISNet [68] where EISNet also utilized unlabeled samples during training. In the SSL setting, we compare our method with traditional methods like MeanTeacher [46], EntMin [49], FixMatch [13], and FreeMatch [23]. In the SSDG setting, we compare our method with StyleMatch [22], and MultiMatch [56] as these two approaches have similar evaluation settings, and official codes are provided. We borrow the results from StyleMatch and MultiMatch in Tables 2 and 3.

**Main results.** Here, full-labels refers to training ERM with all labels in the source domains. Table 4 presents the domain generalization performance of various models in the low-data regime, evaluated on four benchmark datasets: PACS, OfficeHome, VLCS, and miniDomainNet. The baseline "Full-Labels," representing a fully supervised model trained with labeled data, achieves an average accuracy of 79.50% across all datasets in both labeling settings. This serves as a reference point to assess the effectiveness of SSDG methods. Among the SSDG methods, StyleMatch demonstrates reasonable performance, achieving average accuracies of 80.41% and 80.32% for the 10-label and 5-label settings, respectively. However, its reliance on fixed thresholding limits its ability to fully utilize unlabeled data. Similarly, MultiMatch performs slightly worse, with average accuracies of 79.10% and 78.18% for the respective labeling scenarios. In contrast, the proposed method, CAT, achieves superior results across all datasets and labeling conditions. For the 10-label setting, CAT achieves an average accuracy of 82.00%, and for the 5-label setting, it achieves 82.71%, outperforming StyleMatch and MultiMatch by notable margins. CAT’s adaptive thresholding strategy, which incorporates both class-specific and domain-specific information, enables effective utilization of unlabeled data, contributing to its improved performance. When evaluated on individual datasets, CAT consistently achieves the highest accuracy. For instance, on PACS, it achieves 82.95% and 82.71% for the 10-label and 5-label settings, respectively. Similarly, on OfficeHome, CAT records 75.23% and 75.50%. On VLCS, CAT achieves outstanding results of 93.43% and 93.00%, and on miniDomainNet, it obtains 80.10% and 76.19% under the respective label conditions. As shown in the last row of Table 4, all p-values are below 0.0005, indicating that CAT’s improvements are statistically significant at the 0.05 level. This confirms that the observed gains are not due to random chance and highlight the robustness of CAT across diverse domains and data scarcity levels. In summary, the results in Table 4 demonstrate that CAT effectively addresses the challenges of semi-supervised domain generalization in low-data regimes. By leveraging adaptive thresholding, CAT consistently outperforms existing methods across diverse datasets and labeling conditions, highlighting its robustness and practicality for real-world applications.

**Table 4:** Domain generalization results (%) in the low-data regime with a comparison of various models in SSDG settings, evaluated on all datasets. Results are reported as mean ± standard deviation over 5 random seeds. Here, *u* denotes utilization of unlabeled data. Paired t-tests were conducted between CAT and other baselines, with p-values shown in the last row.

Model	*u*	# labels: 10 per class					# labels: 5 per class				
		PACS	OfficeHome	VLCS	miniDomainNet	Avg	PACS	OfficeHome	VLCS	miniDomainNet	Avg
Full-Labels	-	79.50 ± 0.3	64.70 ± 0.4	95.96 ± 0.2	69.20 ± 0.4	79.50 ± 0.3	79.50 ± 0.3	64.70 ± 0.3	95.96 ± 0.4	69.20 ± 0.4	79.50 ± 0.4
**Semi-Supervised Domain Generalization Methods**											
StyleMatch	✓	79.43 ± 0.4	73.75 ± 0.6	90.04 ± 0.3	78.40 ± 0.2	80.41 ± 0.4	78.54 ± 0.1	74.44 ± 0.3	89.25 ± 0.2	79.06 ± 0.3	80.32 ± 0.2
MultiMatch	✓	80.69 ± 0.5	70.44 ± 0.3	90.48 ± 0.4	74.79 ± 0.3	79.10 ± 0.4	79.54 ± 0.2	71.26 ± 0.1	88.00 ± 0.2	73.91 ± 0.3	78.18 ± 0.2
CAT (Ours)	✓	**82.95 ± 0.1**	**75.23 ± 0.2**	**93.43 ± 0.2**	**80.10 ± 0.2**	**82.00 ± 0.2**	**82.71 ± 0.1**	**75.50 ± 0.3**	**93.00 ± 0.1**	**76.19 ± 0.2**	**82.71 ± 0.2**
*p*-value		0.0001	0.0002	0.0003	0.0002	0.0002	0.0002	0.0003	0.0004	0.0003	0.0003

**Results on PACS.** Table 5 provides a detailed comparison of model performance on the PACS dataset in a low-data regime. The Full-Labels model, trained with all labeled data, serves as the upper bound, achieving an average accuracy of 79.50% across both settings. Among the DG methods, which generalize across domains without leveraging unlabeled data, models like Vanilla, CrossGrad, and RSC perform moderately, with RSC achieving an average accuracy of 63.96% (10 labels) and 57.31% (5 labels). EISNet, which does use unlabeled data, shows better performance, reaching 67.18% and 62.04% average accuracies for the two setups, respectively. SSL methods, which utilize unlabeled data to improve performance, generally outperform DG methods. Notable among them are EntMin and FixMatch, with the latter achieving an average accuracy of 75.57% (10 labels) and 70.87% (5 labels). However, FreeMatch exhibits suboptimal adaptation, performing significantly worse with average accuracies of 57.13% and 42.75%, respectively. The SSDG methods, which combine the strengths of DG and SSL, deliver the best results. The proposed CAT (Ours) model achieves state-of-the-art performance, with an average accuracy of 82.95% in the 10-label setting and 82.71% in the 5-label setting. This represents significant improvements over the next-best method, StyleMatch, by 2.54% and 2.39%, respectively. These results underscore the effectiveness of CAT in leveraging both labeled and unlabeled data to handle domain shifts and achieve robust generalization. In summary, the table demonstrates that while DG methods struggle without unlabeled data and SSL methods falter under domain shifts, SSDG methods, particularly CAT, excel by addressing both challenges, achieving superior performance even in extreme low-data scenarios. We also show that CAT’s improvements are statistically significant at the p-value 0.05 level.

**Table 5:** Domain generalization results (%) in the low-data regime with a comparison of various models in different settings (fully labeled, DG, SSL, and SSDG), evaluated on PACS (Photo: P, Art: A, Cartoon: C, and Sketch: S). Here, *u* means utilization of unlabeled data. Results are reported as mean ± standard deviation over 5 random seeds.

Model	*u*	# labels: 10 per class (210 labels)					# labels: 5 per class (105 labels)				
		A	C	P	S	Avg	A	C	P	S	Avg
Full-Labels	-	76.95 ± 0.3	75.90 ± 0.4	95.96 ± 0.6	69.20 ± 0.5	79.50 ± 0.5	76.95 ± 0.6	75.90 ± 0.3	95.96 ± 0.2	69.20 ± 0.5	79.50 ± 0.4
**Domain Generalization Methods**											
Vanilla	✘	63.09 ± 0.4	58.49 ± 0.2	86.56 ± 0.5	45.56 ± 0.3	63.42 ± 0.3	56.71 ± 0.3	53.87 ± 0.4	71.87 ± 0.6	36.96 ± 0.5	54.84 ± 0.4
CrossGrad	✘	62.56 ± 0.3	58.92 ± 0.4	88.41 ± 0.7	44.11 ± 0.2	62.85 ± 0.4	56.29 ± 0.3	53.82 ± 0.4	70.85 ± 0.3	38.52 ± 0.4	54.87 ± 0.3
DDAIG	✘	61.95 ± 0.2	58.74 ± 0.5	84.44 ± 0.5	47.44 ± 0.4	63.64 ± 0.4	56.12 ± 0.3	52.30 ± 0.3	73.68 ± 0.6	38.71 ± 0.2	55.20 ± 0.3
RSC	✘	65.13 ± 0.3	56.65 ± 0.5	86.18 ± 0.4	47.90 ± 0.4	63.96 ± 0.4	58.38 ± 0.2	52.32 ± 0.6	80.42 ± 0.7	40.11 ± 0.2	57.31 ± 0.4
EISNet	✓	66.84 ± 0.2	61.33 ± 0.3	89.36 ± 0.5	53.88 ± 0.6	67.18 ± 0.4	62.08 ± 0.2	54.75 ± 0.3	85.96 ± 0.4	48.60 ± 0.2	62.04 ± 0.3
**Semi-Supervised Learning Methods**											
MeanTeacher	✓	62.41 ± 0.1	57.94 ± 0.2	85.15 ± 0.1	46.66 ± 0.1	63.49 ± 0.1	56.00 ± 0.2	52.64 ± 0.3	73.54 ± 0.1	36.97 ± 0.2	54.79 ± 0.2
EntMin	✓	72.77 ± 0.2	70.55 ± 0.3	89.39 ± 0.4	54.38 ± 0.3	71.77 ± 0.2	67.55 ± 0.2	64.72 ± 0.3	85.33 ± 0.3	49.05 ± 0.2	66.66 ± 0.3
FixMatch	✓	71.80 ± 0.3	68.93 ± 0.1	87.79 ± 0.2	73.75 ± 0.2	75.57 ± 0.2	64.96 ± 0.1	63.62 ± 0.3	83.23 ± 0.2	69.68 ± 0.3	70.87 ± 0.2
FreeMatch	✓	48.44 ± 0.1	60.79 ± 0.1	66.04 ± 0.2	53.23 ± 0.1	57.13 ± 0.1	23.83 ± 0.4	37.28 ± 0.2	61.80 ± 0.2	48.09 ± 0.1	42.75 ± 0.2
**Semi-Supervised Domain Generalization Methods**											
StyleMatch	✓	79.43 ± 0.3	73.75 ± 0.4	90.04 ± 0.2	78.40 ± 0.4	80.41 ± 0.5	78.54 ± 0.3	74.44 ± 0.2	89.25 ± 0.3	79.06 ± 0.3	80.32 ± 0.3
MultiMatch	✓	80.69 ± 0.4	70.44 ± 0.5	90.48 ± 0.3	74.79 ± 0.4	79.10 ± 0.4	79.54 ± 0.3	71.26 ± 0.3	88.00 ± 0.2	73.91 ± 0.1	78.18 ± 0.2
CAT (Ours)	✓	**83.04 ± 0.3**	**75.23 ± 0.2**	**93.43 ± 0.1**	**80.10 ± 0.2**	**82.95 ± 0.1**	**82.83 ± 0.2**	**75.50 ± 0.4**	**93.00 ± 0.4**	**76.19 ± 0.3**	**82.71 ± 0.3**
*p*-value		0.0002	0.0001	0.0003	0.0002	0.0002	0.0001	0.0004	0.0004	0.0002	0.0003

**Results on OfficeHome.** Table 6 provides a detailed comparison of model performance on the OfficeHome dataset in a low-data regime, evaluating models across various experimental settings (e.g. Full labels, DG, SSL, SSDG). The Full-Labels model, trained with fully labeled data, serves as the upper bound, achieving an average accuracy of 64.70% across domains. Among the DG methods, which generalize across domains without using unlabeled data, models such as Vanilla, CrossGrad, and RSC achieve average accuracies of around 57–58% in the 10-label setting and 52–53% in the 5-label setting. RSC and EISNet show slightly better performance due to their enhanced domain generalization capabilities. In contrast, SSL methods like MeanTeacher, EntMin, and FixMatch, which utilize both labeled and unlabeled data, outperform DG methods. For instance, FixMatch+RSC, which combines SSL and domain generalization, achieves average accuracies of 58.88% with 10-labels and 53.91% with 5-labels. On the other hand, SSDG methods, which integrate SSL and DG capabilities, deliver the highest performance across all metrics. Notably, the proposed CAT (Ours) model outperforms all other approaches, achieving an average accuracy of 65.04% in the 10-label setting and 61.71% in the 5-label setting. These results surpass the next-best SSDG method (MultiMatch) by 4.85% and 3.56%, respectively. The significant improvements of CAT highlight its ability to effectively leverage both labeled and unlabeled data while addressing domain shifts. In summary, the results demonstrate that DG methods effectively generalize across domains but fall short without access to unlabeled data. SSL methods improve performance by utilizing unlabeled data but do not account for domain shifts. SSDG methods, particularly CAT, combine the strengths of both approaches, achieving superior generalization and robustness in low-data scenarios. We also show that CAT’s improvements are statistically significant at the p-value 0.05 level.

**Table 6:** Domain generalization results (%) in the low-data regime with a comparison of various models in different settings (fully labeled, DG, SSL, and SSDG), evaluated on OfficeHome (Art: A, Clipart: C, Product: P, and Real-World: R). Here, *u* means utilization of unlabeled data. Results are reported as mean ± standard deviation over 5 runs.

Model	*u*	# labels: 10 per class (1950 labels)					# labels: 5 per class (975 labels)				
		A	C	P	R	Avg	A	C	P	R	Avg
Full-Labels	-	58.88 ± 0.1	49.42 ± 0.2	74.30 ± 0.4	76.21 ± 0.2	64.70 ± 0.2	58.88 ± 0.2	49.42 ± 0.3	74.30 ± 0.2	76.21 ± 0.1	64.70 ± 0.2
**Domain Generalization Methods**											
Vanilla	✘	50.11 ± 0.1	43.50 ± 0.3	61.11 ± 0.6	69.65 ± 0.4	57.09 ± 0.3	45.76 ± 0.3	39.97 ± 0.3	60.04 ± 0.2	63.77 ± 0.1	52.38 ± 0.2
CrossGrad	✘	50.32 ± 0.3	43.27 ± 0.2	61.56 ± 0.1	69.77 ± 0.2	57.23 ± 0.3	45.89 ± 0.3	40.17 ± 0.2	60.63 ± 0.3	63.64 ± 0.2	52.54 ± 0.3
DDAIG	✘	49.65 ± 0.2	42.52 ± 0.3	63.54 ± 0.2	67.89 ± 0.1	55.65 ± 0.2	45.33 ± 0.4	39.82 ± 0.3	62.33 ± 0.2	62.77 ± 0.4	52.06 ± 0.3
RSC	✘	49.65 ± 0.1	42.33 ± 0.4	64.88 ± 0.5	69.26 ± 0.4	56.03 ± 0.4	46.09 ± 0.3	39.59 ± 0.2	63.77 ± 0.2	63.86 ± 0.3	53.08 ± 0.2
EISNet	✘	51.16 ± 0.2	43.33 ± 0.1	64.72 ± 0.4	65.89 ± 0.3	56.28 ± 0.3	47.32 ± 0.1	40.47 ± 0.4	63.84 ± 0.4	62.32 ± 0.2	53.23 ± 0.3
**Semi-Supervised Learning Methods**											
MeanTeacher	✓	49.92 ± 0.1	43.42 ± 0.2	64.61 ± 0.2	68.79 ± 0.3	56.69 ± 0.2	45.96 ± 0.1	39.15 ± 0.2	59.18 ± 0.3	62.98 ± 0.2	51.49 ± 0.2
EntMin	✓	51.44 ± 0.6	44.92 ± 0.2	66.85 ± 0.5	70.52 ± 0.3	58.45 ± 0.4	48.11 ± 0.4	41.72 ± 0.5	62.41 ± 0.6	63.19 ± 0.2	53.36 ± 0.4
FixMatch	✓	50.36 ± 0.3	49.70 ± 0.3	63.93 ± 0.4	67.56 ± 0.2	57.89 ± 0.2	47.88 ± 0.4	40.50 ± 0.3	62.06 ± 0.2	62.77 ± 0.1	53.30 ± 0.2
FixMatch+RSC	✓	51.49 ± 0.2	43.77 ± 0.1	66.83 ± 0.5	68.29 ± 0.6	58.88 ± 0.4	48.05 ± 0.3	40.66 ± 0.2	63.82 ± 0.3	62.82 ± 0.3	53.91 ± 0.3
**Semi-Supervised Domain Generalization Methods**											
StyleMatch	✓	52.82 ± 0.6	51.60 ± 0.5	65.31 ± 0.4	68.61 ± 0.5	59.59 ± 0.5	51.53 ± 0.3	50.00 ± 0.4	60.88 ± 0.2	64.47 ± 0.3	56.72 ± 0.3
MultiMatch	✓	52.91 ± 0.4	50.63 ± 0.5	66.67 ± 0.4	70.55 ± 0.4	60.19 ± 0.3	51.80 ± 0.3	49.02 ± 0.2	64.16 ± 0.3	67.60 ± 0.4	58.15 ± 0.2
CAT (Ours)	✓	**57.28 ± 0.4**	**54.13 ± 0.5**	**73.10 ± 0.3**	**75.67 ± 0.2**	**65.04 ± 0.3**	**55.73 ± 0.3**	**51.29 ± 0.4**	**69.25 ± 0.3**	**70.57 ± 0.4**	**61.71 ± 0.3**
*p*-value		0.0002	0.0003	0.0002	0.0001	0.0002	0.0001	0.0002	0.0003	0.0002	0.0002

**Results on miniDomainNet.** Table 7 summarizes the results of different models evaluated on the miniDomainNet dataset under a low-data regime. The Full-Labels model achieves the best performance, setting an upper limit with average accuracies of 68.18% in the 10-label setting and 66.27% in the 5-label setting. These results highlight the optimal scenario where full supervision is available. Among SSDG methods, StyleMatch achieves average accuracies of 63.32% (10-label) and 61.26% (5-label), demonstrating its ability to leverage unlabeled data to address domain generalization. However, it is surpassed by MultiMatch, which improves the average accuracies to 64.55% and 63.70% for the two settings, respectively, indicating stronger capabilities to handle domain shifts. Our model significantly outperforms the other SSDG methods, achieving state-of-the-art average accuracies of 67.71% in the 10-label setting and 66.32% in the 5-label setting. These results closely approach the performance of the fully supervised Full-Labels model, demonstrating the model’s effectiveness in leveraging both labeled and unlabeled data. Compared to StyleMatch, CAT achieves a +4.39% improvement in the 10-label setting and a +5.06% improvement in the 5-label setting, while also outperforming MultiMatch by +3.16% and +2.62%, respectively. In conclusion, the results highlight the superior performance of CAT in addressing the challenges of domain generalization and limited labeled data. Its ability to achieve results comparable to the Full-Labels model makes it a robust solution for real-world low-data scenarios on the miniDomainNet dataset. We also show that CAT’s improvements are statistically significant at the p-value 0.05 level.

**Table 7:** Domain generalization results (%) in the low-data regime with a comparison of various models in different settings (fully labeled, DG, SSL, and SSDG), evaluated on miniDomainNet (Clipart: C, Painting: P, Real: R, and Sketch: S). Here, *u* means utilization of unlabeled data. Results are reported as mean ± standard deviation over 5 runs.

Model	*u*	# labels: 10 per class (3780 labels)					# labels: 5 per class (1890 labels)				
		C	P	R	S	Avg	C	P	R	S	Avg
Full-Labels	-	68.29 ± 0.2	67.13 ± 0.3	69.78 ± 0.4	67.50 ± 0.4	68.18 ± 0.3	66.14 ± 0.1	63.56 ± 0.4	70.10 ± 0.3	65.28 ± 0.2	66.27 ± 0.2
**Semi-Supervised Domain Generalization Methods**											
StyleMatch	✓	61.98 ± 0.3	60.28 ± 0.4	66.23 ± 0.4	64.80 ± 0.3	63.32 ± 0.4	60.25 ± 0.3	58.19 ± 0.4	63.20 ± 0.3	63.41 ± 0.2	61.26 ± 0.3
MultiMatch	✓	63.82 ± 0.4	61.29 ± 0.8	66.90 ± 0.4	66.19 ± 0.4	64.55 ± 0.5	62.41 ± 0.3	60.41 ± 0.6	65.92 ± 0.4	66.07 ± 0.5	63.70 ± 0.3
CAT (Ours)	✓	**66.97 ± 0.5**	**66.10 ± 0.3**	**70.21 ± 0.4**	**67.54 ± 0.3**	**67.71 ± 0.4**	**65.89 ± 0.3**	**64.26 ± 0.3**	**69.25 ± 0.2**	**65.87 ± 0.5**	**66.32 ± 0.3**
*p*-value		0.0001	0.0003	0.0003	0.0001	0.0002	0.0002	0.0001	0.0003	0.0002	0.0002

**Inference time.** We assess the runtime efficiency of our proposed CAT and StyleMatch on the PACS dataset with a ResNet-18 backbone. We compared the models in RTX 3090 GPU with a batch size of 64. In Table 8, we see that CAT has a lower inference time and total test time. This shows our framework is more compatible with low-latency or real-time applications.

**Table 8:** Inference time comparison on the PACS dataset using ResNet-18 backbone and NVIDIA RTX 3090 GPU.

Method	Backbone	Inference Time (ms)	Total Test Time (s)
StyleMatch	ResNet-18	4.7	3.4
CAT (Ours)	ResNet-18	4.3	3.1

### 5.2 Ablation studies

**Effectiveness of different backbones.** In Table 9, We compare both ResNet-18 and ResNet-50, CAT consistently outperforms both StyleMatch and MultiMatch across all domains and label settings. Specifically, when using ResNet-18, CAT achieves average performance scores of 82.95% and 82.71% for the 10-label and 5-label configurations, respectively. With ResNet-50, CAT performs even better, reaching average scores of 85.29% and 85.05% in the same two label settings. In comparison, StyleMatch shows competitive performance, but CAT consistently surpasses it, especially in the 10-label settings. For instance, with ResNet-50 and 10 labels per class, StyleMatch achieves an average score of 82.45%, while CAT achieves a significantly higher average of 85.29%. MultiMatch, while also competitive, does not match the performance of CAT in either backbone setting. Overall, the results suggest that the proposed CAT method is more effective in SSDG tasks than StyleMatch and MultiMatch. Moreover, the deeper ResNet-50 backbone outperforms the ResNet-18 backbone across both label configurations, indicating that a more complex network architecture benefits the performance of the models in this task.

**Table 9:** Backbone comparison of ResNet-18 and ResNet-50 in SSDG settings on PACS. Results are reported as mean ± standard deviation over 5 random seeds.

Model	*u*	# labels: 10 per class (210 labels)					# labels: 5 per class (105 labels)				
		A	C	P	S	Avg	A	C	P	S	Avg
**ResNet-18**											
StyleMatch	✓	79.43 ± 0.2	73.75 ± 0.3	90.04 ± 0.3	78.40 ± 0.4	80.41 ± 0.3	78.54 ± 0.3	74.44 ± 0.4	89.25 ± 0.2	79.06 ± 0.3	80.32 ± 0.3
MultiMatch	✓	80.69 ± 0.1	70.44 ± 0.5	90.48 ± 0.3	74.79 ± 0.3	79.10 ± 0.3	79.54 ± 0.4	71.26 ± 0.5	88.00 ± 0.3	73.91 ± 0.3	78.18 ± 0.3
CAT (Ours)	✓	**83.04 ± 0.3**	**75.23 ± 0.2**	**93.43 ± 0.2**	**80.10 ± 0.3**	**82.95 ± 0.2**	**82.83 ± 0.3**	**75.50 ± 0.2**	**93.00 ± 0.4**	**76.19 ± 0.2**	**82.71 ± 0.2**
**ResNet-50**											
StyleMatch	✓	81.72 ± 0.2	76.19 ± 0.3	92.58 ± 0.3	80.04 ± 0.2	82.45 ± 0.3	80.18 ± 0.2	76.58 ± 0.3	91.09 ± 0.1	81.42 ± 0.3	82.96 ± 0.2
MultiMatch	✓	83.23 ± 0.2	72.48 ± 0.3	92.87 ± 0.3	77.23 ± 0.2	81.49 ± 0.2	81.59 ± 0.3	73.63 ± 0.2	90.30 ± 0.3	76.45 ± 0.2	80.59 ± 0.3
CAT (Ours)	✓	**85.38 ± 0.2**	**77.57 ± 0.1**	**95.77 ± 0.3**	**82.44 ± 0.2**	**85.29 ± 0.2**	**85.17 ± 0.3**	**77.84 ± 0.2**	**95.34 ± 0.3**	**78.53 ± 0.3**	**85.05 ± 0.2**

**Fig 3:**
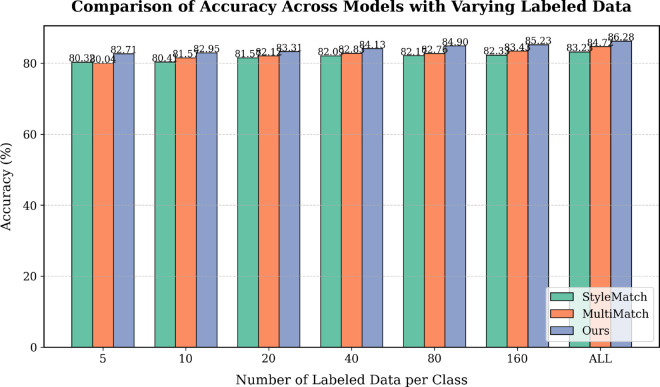
Comparison between our method with StyleMatch and MultiMatch in different label settings.

**Effect of different numbers of labels.** In Fig 3, we conduct a comparison with different sets of label data to validate the performance of our method. We compare with two SSDG methods, such as StyleMatch, and MultiMatch. In every label set, our method outperforms both StyleMatch and MultiMatch. In all label settings, our method can improve performance by 1.5% than MultiMatch, which is better 1.5% better than StyleMatch. Hence, these results demonstrate its effectiveness even in a fully supervised setting.

**Effect of different numbers of source domains.** In Table 10, we examine the impact of the number of sources (*K*) on the performance of three models—FixMatch, StyleMatch, and CAT (the proposed method)—on the PACS dataset, under two settings of label availability: 10 labels per class and 5 labels per class. The results, reported as accuracy percentages, highlight the influence of *K* (number of source domains) and the availability of labeled data on the models’ performance. The results reveal that increasing the number of sources (*K*) consistently improves accuracy across all models. For instance, FixMatch shows notable improvements as *K* increases from 1 to 3, but it lags behind StyleMatch and CAT in every configuration. StyleMatch demonstrates better utilization of domain information, consistently outperforming FixMatch across both label regimes. However, CAT significantly surpasses both FixMatch and StyleMatch in all scenarios, indicating its superior capability in leveraging both labeled and unlabeled data for domain generalization. With 10 labels per class, CAT achieves the highest accuracy, with 61.32% for *K* = 1, 78.92% for *K* = 2, and 82.95% for *K* = 3. Even in the low-data regime of 5 labels per class, CAT maintains its dominance, achieving 57.64% for *K* = 1, 74.26% for *K* = 2, and 82.71% for *K* = 3. These results highlight the model’s robustness and scalability, particularly as the number of source domains (*K*) increases. In summary, the findings demonstrate that CAT consistently outperforms FixMatch and StyleMatch, especially as the number of sources grows. Furthermore, it shows remarkable robustness in low-data scenarios, confirming its effectiveness in domain generalization tasks under varying conditions of labeled data availability.

**Table 10:** Impact on the number of sources (*K*) on the PACS dataset with varying label availability: 10 labels per class and 5 labels per class.

Model	10 labels			5 labels		
	*K* = 1	*K* = 2	*K* = 3	*K* = 1	*K* = 2	*K* = 3
FixMatch	53.55	71.42	77.12	49.91	68.52	74.94
StyleMatch	57.29	74.50	80.41	52.24	71.95	80.32
CAT (Ours)	**61.32**	**78.92**	**82.95**	**57.64**	**74.26**	**82.71**

## 6 Conclusion

In this work, we explore the challenging area of semi-supervised domain generalization (SSDG) to handle domain shifts under a low-data regime. In recent years, SSDG has become a more practical solution for many real-world applications. Hence, we propose **CAT**, an SSDG method that addresses the limitations of existing approaches by leveraging adaptive thresholding and noisy label refinement techniques to generate reliable pseudo labels and enhance generalization. By employing both global and local adaptive thresholds, our method ensures improved class diversity and dynamic confidence management in pseudo label generation. Additionally, the integration of supervised contrastive learning with refined pseudo-labels enables the model to capture domain-invariant representations effectively. Experimental results demonstrate the effectiveness of our method as an SSDG solution.

In future work, researchers can focus on the continual adaptation of domains under distribution change and label scarcity. Secondly, uncertainty-guided model generalization under severe domain shift can be explored. Thirdly, lightweight integration of vision language models in SSDG settings. And finally, generalization under the open-set setting is still an open challenge in SSDG settings.

## References

[1] Sanchez J, Deschaud JE, Goulette F. Domain generalization of 3D semantic segmentation in autonomous driving. In: Proceedings of the IEEE/CVF International Conference on Computer Vision. 2023. p. 18077–87.

[2] Yang F, Chen H, He Y, Zhao S, Zhang C, Ni K, et al. Geometry-guided domain generalization for monocular 3d object detection. In: Proceedings of the AAAI Conference on Artificial Intelligence. vol. 38; 2024. p. 6467–76.

[3] Chen L, Zhang Y, Song Y, Van Den Hengel A, Liu L. Domain generalization via rationale invariance. In: 2023 IEEE/CVF International Conference on Computer Vision (ICCV). 2023. p. 1751–60. 10.1109/iccv51070.2023.00168

[4] Learning SS. Semi-supervised learning. CSZ. 2006;5(2).

[5] Hady MFA, Schwenker F. Semi-supervised learning. Handbook on neural information processing. 2013. p. 215–39.

[6] Zhu X, Goldberg AB. Introduction to semi-supervised learning. Springer; 2022.

[7] Berthelot D, Carlini N, Goodfellow I, Papernot N, Oliver A, Raffel CA. Mixmatch: A holistic approach to semi-supervised learning. Advances in Neural Information Processing Systems. 2019;32.

[8] Yang X, Song Z, King I, Xu Z. A survey on deep semi-supervised learning. IEEE Trans Knowl Data Eng. 2023;35(9):8934–54. doi: 10.1109/tkde.2022.3220219

[9] Lee DH, et al. Pseudo-label: The simple and efficient semi-supervised learning method for deep neural networks. In: Workshop on challenges in representation learning, ICML, Atlanta, 2013. p. 896.

[10] Bachman P, Alsharif O, Precup D. Learning with pseudo-ensembles. Advances in Neural Information Processing Systems. 2014;27.

[11] Cascante-Bonilla P, Tan F, Qi Y, Ordonez V. Curriculum labeling: Revisiting pseudo-labeling for semi-supervised learning. In: Proceedings of the AAAI Conference on Artificial Intelligence. 2021. p. 6912–20.

[12] Abuduweili A, Li X, Shi H, Xu CZ, Dou D. Adaptive consistency regularization for semi-supervised transfer learning. In: Proceedings of the IEEE/CVF Conference on Computer Vision and Pattern Recognition. 2021. p. 6923–32.

[13] Sohn K, Berthelot D, Carlini N, Zhang Z, Zhang H, Raffel CA. Fixmatch: Simplifying semi-supervised learning with consistency and confidence. Advances in Neural Information Processing Systems. 2020;33:596–608.

[14] Verma V, Kawaguchi K, Lamb A, Kannala J, Solin A, Bengio Y, et al. Interpolation consistency training for semi-supervised learning. Neural Netw. 2022;145:90–106. doi: 10.1016/j.neunet.2021.10.008 34735894

[15] Zhang M, Marklund H, Dhawan N, Gupta A, Levine S, Finn C. Adaptive risk minimization: learning to adapt to domain shift. Advances in Neural Information Processing Systems. 2021;34:23664–78.

[16] Stacke K, Eilertsen G, Unger J, Lundstrom C. Measuring domain shift for deep learning in histopathology. IEEE J Biomed Health Inform. 2021;25(2):325–36. doi: 10.1109/JBHI.2020.3032060 33085623

[17] Chen Y, Wei C, Kumar A, Ma T. Self-training avoids using spurious features under domain shift. Advances in Neural Information Processing Systems. 2020;33:21061–71.

[18] Zhou K, Liu Z, Qiao Y, Xiang T, Loy CC. Domain generalization: a survey. IEEE Trans Pattern Anal Mach Intell. 2023;45(4):4396–415. doi: 10.1109/TPAMI.2022.3195549 35914036

[19] Wang J, Lan C, Liu C, Ouyang Y, Qin T, Lu W, et al. Generalizing to unseen domains: a survey on domain generalization. IEEE Trans Knowl Data Eng. 2022;1.doi: 10.1109/tkde.2022.3178128

[20] Zhou K, Yang Y, Qiao Y, Xiang T. Domain adaptive ensemble learning. IEEE Trans Image Process. 2021;30:8008–18. doi: 10.1109/TIP.2021.3112012 34534081

[21] Li D, Yang Y, Song YZ, Hospedales TM. Deeper, broader and artier domain generalization. In: Proceedings of the IEEE International Conference on Computer Vision. 2017. p. 5542–50.

[22] Zhou K, Loy CC, Liu Z. Semi-supervised domain generalization with stochastic StyleMatch. Int J Comput Vis. 2023;131(9):2377–87. doi: 10.1007/s11263-023-01821-x

[23] Wang Y, Chen H, Heng Q, Hou W, Fan Y, Wu Z. Freematch: self-adaptive thresholding for semi-supervised learning. arXiv preprint 2022. doi: arXiv:2205.07246

[24] Guo LZ, Li YF. Class-imbalanced semi-supervised learning with adaptive thresholding. In: International Conference on Machine Learning. PMLR; 2022. p. 8082–94.

[25] Xu Y, Shang L, Ye J, Qian Q, Li YF, Sun B. Dash: Semi-supervised learning with dynamic thresholding. In: International Conference on Machine Learning. PMLR; 2021. p. 11525–36.

[26] Berthelot D, Roelofs R, Sohn K, Carlini N, Kurakin A. Adamatch: a unified approach to semi-supervised learning and domain adaptation. 2021. https://arxiv.org/abs/2106.04732

[27] Yao X, Bai Y, Zhang X, Zhang Y, Sun Q, Chen R. PCL: Proxy-based contrastive learning for domain generalization. In: Proceedings of the IEEE/CVF Conference on Computer Vision and Pattern Recognition. 2022. p. 7097–107.

[28] Muandet K, Balduzzi D, Schölkopf B. Domain generalization via invariant feature representation. In: International Conference on Machine Learning. PMLR; 2013. p. 10–8.

[29] Li H, Wang Y, Wan R, Wang S, Li TQ, Kot A. Domain generalization for medical imaging classification with linear-dependency regularization. Advances in neural information processing systems. 2020;33:3118–29.

[30] Li Y, Gong M, Tian X, Liu T, Tao D. Domain generalization via conditional invariant representations. AAAI. 2018;32(1). doi: 10.1609/aaai.v32i1.11682

[31] Balaji Y, Sankaranarayanan S, Chellappa R. Metareg: towards domain generalization using meta-regularization. Advances in neural information processing systems. 2018;31.

[32] Li Y, Yang Y, Zhou W, Hospedales T. Feature-critic networks for heterogeneous domain generalization. In: International Conference on Machine Learning. PMLR; 2019. p. 3915–24.

[33] Ganin Y, Ustinova E, Ajakan H, Germain P, Larochelle H, Laviolette F. Domain-adversarial training of neural networks. Journal of Machine Learning Research. 2016;17(59):1–35.

[34] Zhou K, Yang Y, Qiao Y, Xiang T. Mixstyle neural networks for domain generalization and adaptation. International Journal of Computer Vision. 2024;132(3):822–36.

[35] Mancini M, Akata Z, Ricci E, Caputo B. Towards recognizing unseen categories in unseen domains. In: European Conference on Computer Vision. Springer; 2020. p. 466–83.

[36] Xu Z, Liu D, Yang J, Raffel C, Niethammer M. Robust and generalizable visual representation learning via random convolutions. arXiv preprint 2020. https://arxiv.org/abs/2007.13003

[37] Ding Z, Fu Y. Deep domain generalization with structured low-rank constraint. IEEE Trans Image Process. 2018;27(1):304–13. doi: 10.1109/TIP.2017.2758199 28976316

[38] D’Innocente A, Caputo B. Domain generalization with domain-specific aggregation modules. In: Pattern Recognition: 40th German Conference, GCPR 2018, Stuttgart, Germany, October 9-12, 2018, Proceedings 40. Springer; 2019. p. 187–98.

[39] Seo S, Suh Y, Kim D, Kim G, Han J, Han B. Learning to optimize domain specific normalization for domain generalization. In: Computer Vision–ECCV 2020 : 16th European Conference, Glasgow, UK, August 23–28, 2020, Proceedings, Part XXII 16. Springer; 2020. p. 68–83.

[40] Mancini M, Bulo SR, Caputo B, Ricci E. Robust place categorization with deep domain generalization. IEEE Robotics and Automation Letters. 2018;3(3):2093–100.

[41] Noroozi M, Favaro P. Unsupervised learning of visual representations by solving jigsaw puzzles. In: European Conference on Computer Vision. Springer; 2016. p. 69–84.

[42] Gidaris S, Singh P, Komodakis N. Unsupervised representation learning by predicting image rotations. arXiv preprint 2018. https://arxiv.org/abs/1803.07728

[43] Kim D, Yoo Y, Park S, Kim J, Lee J. Selfreg: self-supervised contrastive regularization for domain generalization. In: Proceedings of the IEEE/CVF International Conference on Computer Vision. 2021. p. 9619–28.

[44] Cha J, Lee K, Park S, Chun S. Domain generalization by mutual-information regularization with pre-trained models. In: European Conference on Computer Vision. Springer; 2022. p. 440–57.

[45] Tai KS, Bailis PD, Valiant G. Sinkhorn label allocation: Semi-supervised classification via annealed self-training. In: International Conference on Machine Learning. PMLR; 2021. p. 10065–75.

[46] Tarvainen A, Valpola H. Mean teachers are better role models: weight-averaged consistency targets improve semi-supervised deep learning results. Advances in neural information processing systems. 2017;30.

[47] Ke Z, Wang D, Yan Q, Ren J, Lau RW. Dual student: breaking the limits of the teacher in semi-supervised learning. In: Proceedings of the IEEE/CVF International Conference on Computer Vision; 2019. p. 6728–36.

[48] Luo Y, Zhu J, Li M, Ren Y, Zhang B. Smooth neighbors on teacher graphs for semi-supervised learning. In: Proceedings of the IEEE Conference on Computer Vision and Pattern Recognition; 2018. p. 8896–905.

[49] Grandvalet Y, Bengio Y. Semi-supervised learning by entropy minimization. Advances in Neural Information Processing Systems. 2004;17.

[50] Xie Q, Dai Z, Hovy E, Luong T, Le Q. Unsupervised data augmentation for consistency training. Advances in Neural Information Processing Systems. 2020;33:6256–68.

[51] Chen B, Jiang J, Wang X, Wan P, Wang J, Long M. Debiased self-training for semi-supervised learning. Advances in Neural Information Processing Systems. 2022;35:32424–37.

[52] Zhao Z, Zhou L, Wang L, Shi Y, Gao Y. LaSSL: label-guided self-training for semi-supervised learning. AAAI. 2022;36(8):9208–16. doi: 10.1609/aaai.v36i8.20907

[53] Wei C, Sohn K, Mellina C, Yuille A, Yang F. Crest: a class-rebalancing self-training framework for imbalanced semi-supervised learning. In: Proceedings of the IEEE/CVF Conference on Computer Vision and Pattern Recognition. 2021. p. 10857–66.

[54] Amini M-R, Feofanov V, Pauletto L, Hadjadj L, Devijver É, Maximov Y. Self-training: a survey. Neurocomputing. 2025;616:128904. doi: 10.1016/j.neucom.2024.128904

[55] Zhang L, Li JF, Wang W. Semi-supervised domain generalization with known and unknown classes. Advances in Neural Information Processing Systems. 2023;36:28735–47.

[56] Qi L, Yang H, Shi Y, Geng X. MultiMatch: multi-task learning for semi-supervised domain generalization. ACM Trans Multimedia Comput Commun Appl. 2024;20(6):1–21. doi: 10.1145/3648680

[57] Jayanaga Galappaththige C, Izzo Z, He X, Zhou H, Haris Khan M. Domain-guided weight modulation for semi-supervised domain generalization. arXiv preprint 2024. p. 2409.

[58] Cubuk ED, Zoph B, Shlens J, Le QV. Randaugment: practical automated data augmentation with a reduced search space. In: Proceedings of the IEEE/CVF Conference on Computer Vision and Pattern Recognition Workshops; 2020. p. 702–3.

[59] Khosla P, Teterwak P, Wang C, Sarna A, Tian Y, Isola P. Supervised contrastive learning. Advances in Neural Information Processing Systems. 2020;33:18661–73.

[60] Ortego D, Arazo E, Albert P, O’Connor NE, McGuinness K. Multi-objective interpolation training for robustness to label noise. In: Proceedings of the IEEE/CVF Conference on Computer Vision and Pattern Recognition; 2021. p. 6606–15.

[61] Li S, Xia X, Ge S, Liu T. Selective-supervised contrastive learning with noisy labels. In: 2022 IEEE/CVF Conference on Computer Vision and Pattern Recognition (CVPR). 2022. p. 316–25. 10.1109/cvpr52688.2022.00041

[62] Venkateswara H, Eusebio J, Chakraborty S, Panchanathan S. Deep hashing network for unsupervised domain adaptation. In: Proceedings of the IEEE Conference on Computer Vision and Pattern Recognition; 2017. p. 5018–27.

[63] Torralba A, Efros AA. Unbiased look at dataset bias. In: CVPR 2011 . IEEE; 2011. p. 1521–8.

[64] He K, Zhang X, Ren S, Sun J. Deep residual learning for image recognition. In: Proceedings of the IEEE Conference on Computer Vision and Pattern Recognition. 2016. p. 770–8.

[65] Shankar S, Piratla V, Chakrabarti S, Chaudhuri S, Jyothi P, Sarawagi S. Generalizing across domains via cross-gradient training. arXiv preprint 2018. doi: arXiv:1804.10745

[66] Zhou K, Yang Y, Hospedales T, Xiang T. Deep domain-adversarial image generation for domain generalisation. In: Proceedings of the AAAI Conference on Artificial Intelligence. vol. 34; 2020. p. 13025–32.

[67] Huang Z, Wang H, Xing EP, Huang D. Self-challenging improves cross-domain generalization. In: Computer Vision–ECCV 2020 : 16th European Conference, Glasgow, UK, August 23–28, 2020, Proceedings, Part II 16. Springer; 2020. p. 124–40.

[68] Wang S, Yu L, Li C, Fu CW, Heng PA. Learning from extrinsic and intrinsic supervisions for domain generalization. In: European Conference on Computer Vision. Springer; 2020. p. 159–76.

